# Guidelines for burn rehabilitation in China

**DOI:** 10.1186/s41038-015-0019-3

**Published:** 2015-10-21

**Authors:** Chinese Burn Association, Chinese Association of Burn Surgeons, Ying Cen, Jiake Chai, Huade Chen, Jian Chen, Guanghua Guo, Chunmao Han, Dahai Hu, Jingning Huan, Xiaoyuan Huang, Chiyu Jia, Cecilia WP Li-Tsang, Jianan Li, Zongyu Li, Qun Liu, Yi Liu, Gaoxing Luo, Guozhong Lv, Xihua Niu, Daizhi Peng, Yizhi Peng, Hongyan Qi, Shunzhen Qi, Zhiyong Sheng, Dan Tang, Yibing Wang, Jun Wu, Zhaofan Xia, Weiguo Xie, Hongming Yang, Xianfeng Yi, Lehua Yu, Guoan Zhang, The Chinese Burn Care and Rehabilitation Association

**Affiliations:** 1 Department of Burn and Plastic Surgery, West China School of Medicine, West China Hospital, Sichuan University, Chengdu, Sichuan China; 2 Department of Burn & Plastic Surgery, the First Hospital Affiliated to General Hospital of PLA, Beijing, China; 3 Department of Burns, General Hospital of Guangdong Province, Guangzhou, Guangdong China; 4 State Key Laboratory of Trauma, Burns and Combined Injury, Institute of Burn Research, Southwest Hospital, the Third Military Medical University, Chongqing, China; 5 Department of Burns, the First Affiliated Hospital of Nanchang Univerisity, Research Center of Technology of Wound Repair Engineering in Jiangxi Province, Nanchang, Jiangxi China; 6 Department of Burns and Wound Center, The Second Affiliated Hospital, School of Medicine, Zhejiang University, Hangzhou, Zhejiang China; 7 Department of Burns and Cutaneous Surgery, Xijing Hospital, Fourth Military Medical University, Xi’an, Shanxi China; 8 Department of Burn and Plastic Surgery, Ruijin Hospital, School of Medicine, Shanghai Jiao Tong University, Shanghai, China; 9 Department of Burns and Plastic Surgery, Central South University, Changsha, Hunan China; 10 Plastic Beauty and Burn Repair Center, the 309th Hospital of the Chinese PLA, Beijing, China; 11 Department of Rehabilitation Sciences, the Hong Kong Polytechnic University, Hung Hom, Hong Kong, China; 12 Department of Rehabilitation Medicine, the First Affiliated Hospital of Nanjing Medical University, Nanjing, Jiangsu China; 13 Department of Burns and Plastic Surgery, the Fifth Hospital of Harbin, Harbin, Heilongjiang Province China; 14 Department of Burn and Plastic Surgery, the Fourth Hospital of Tianjin, Burn Institution of Tianjin, Tianjin, China; 15 Burns and Plastic Surgery Center, PLA Lanzhou General Hospital of Lanzhou Command, Lanzhou, Gansu China; 16 Department of Burn Surgery, the Third People’s Hospital of Wuxi, Jiangsu, China; 17 Department of Burn Surgery, the First People’s Hospital of ZhengZhou, Zhengzhou, Henan China; 18 Department of Burn Surgery, Beijing Children’s Hospital, Beijing, China; 19 The Center of Burn and Plastic of Hebei Province, Bethune International Peace Hospital, Shijiazhuang, Hebei China; 20 Guangdong Provincial Work Injury Rehabilitation Center, Guangzhou, Guangdong China; 21 Department of Burns and Plastic Surgery, Provincial Hospital Affiliated to Shandong University, Jinan, Shandong China; 22 Department of Burn Surgery, Changhai Hospital, Second Military Medical University, Shanghai, China; 23 Institute of Burns, Wuhan City Hospital No. 3 & Tongren Hospital of Wuhan University, Wuhan, Hubei China; 24 Department of Rehabilitation Medicine, the Second Affiliated Hospital of Chongqing Medical University, Chongqing, China; 25 Department of Burns, Beijing Jishuitan Hospital, Forth Medical College of Peking University, Beijing, China

**Keywords:** Burn, Rehabilitation, Physical therapy, Occupational Therapy, Scar

## Abstract

Quality of life and functional recovery after burn injury is the final goal of burn care, especially as most of burn patients survive the injury due to advanced medical science. However, dysfunction, disfigurement, contractures, psychological problems and other discomforts due to burns and the consequent scars are common, and physical therapy and occupational therapy provide alternative treatments for these problems of burn patients. This guideline, organized by the Chinese Burn Association and Chinese Association of Burn Surgeons aims to emphasize the importance of team work in burn care and provide a brief introduction of the outlines of physical and occupational therapies during burn treatment, which is suitable for the current medical circumstances of China. It can be used as the start of the tools for burn rehabilitation.

## Background

With the improvement of medical science, wound healing and life saving are no longer the only goal of burn care. The importance of deformity prevention, functional restoration, aesthetic improvement and return to family and society has become more apparent for patients and families as well as burn caregivers.

This preliminary guideline is written on the basis of our nation-wide survey on the current status of burn rehabilitation in China [1]. In addition, we also make references to several practice guidelines for burn rehabilitation from European countries and the U.S. as references [2]. It was considered suitable for the current medical service level in China at the conference of Chinese Burn Association and Chinese Association of Burn Surgeons. This guideline is only a starting point, with revisions and improvements through clinical practices; it will become more comprehensive and practical, and will be of benefit for more burn patients in China as well as other countries.

## Goal setting for burn rehabilitation

Short-term goal: To maintain and gradually increase the range of motion (ROM) in the uninjured and injured areas, to reduce edema and pain, to improve muscle strength and endurance, to prevent contracture, and to minimize scar formation.

Long-term goal: To improve ROM and muscle strength, to further enhance exercise capacity, flexibility and coordination, and to restore the ability of ambulation.

Criteria for discharge: Patients are able to transfer, ambulate, eat, use the toilet, and perform other activities of daily living without or with some assistance.

The ultimate goal: patients can restore their abilities to their pre-injury condition, return to family and society: 1) Independent ADL, studying and working; 2) better aesthetic appearances; and 3) better psychological adaptation [3, 4].

## Concerns of burn rehabilitation

Rehabilitation of burn patients should focus more on the following conditions [5, 6]: 1) Muscle atrophy and reduced muscle strength, endurance, balance and coordination due to immobilization; 2) Reduced ROM caused by deposition of fibrous tissues and adhesion of soft tissue around joints due to immobilization; 3) Anchylosis and deformity caused by hypertrophic scarring or contraction of soft tissues such as scar, tendons, capsules of joints and muscles due to immobilization; 4) Cardiorespiratory reconditioning, hypostatic pneumonia, deep venous thrombosis, and pressure sores due to immobilization; 5) Adjuvant therapies to help the healing of burn wounds, wounds infection control, and limb edema; 6) Abnormal pigmentation caused by burns and disfigurement caused by hypertrophic scarring; 7) Adjuvant therapies to improve symptoms caused by scars and wounds such as paresthesia, pain, itching, and sleep disorder [7]; 8) Decreased ADL, learning and working abilities after injury; 9) Social and psychological disorders caused by burns [8]; 10) Follow-up of patients as outpatients after discharge.

## Scope of burn rehabilitation

Rehabilitation after burns should include the following: 1) Patient and care-giver education on rehabilitation; 2) Rehabilitation assessment [9]; 3) Positioning; 4) Exercises for improving muscle strength, endurance, balance, coordination, and cardiopulmonary function as well as preventing deep venous thrombosis and pressure sores; 5) Active and passive exercises to maintain and improve ROM [10]; 6) Occupational therapy, vocational therapy, and training programs to improve ADLs; 7) Splinting to prevent and ameliorate deformity, and maintain joint function; 8) Physical therapies to promote wound healing and infection control; 9) Physical therapies for contractures of hypertrophic scar, limb swelling, acute and chronic inflammation, pain, and itching; 10) Comprehensive scar treatments such as pressure therapy [11], massage, stretching, splinting, intra-lesional injections of medications, skin care for hypo-pigmentation, hyper-pigmentation, and hyperemia, laser therapy, and techniques of scar camouflage; 11) Medications to alleviate the symptoms such as pain, itching and sleep disorders; 12) Psychological assessment, counseling and therapy [12]; 13) Monitoring and treatments of nutritional disorders and organ functions.

## The team work of burn rehabilitation

### Team members

Rehabilitation of a burn patient requires a team approach. No one can achieve the goal alone [13]. Therefore, a multidisciplinary teamwork model system is advocated and established in different burn care units [14, 15] to meet the common goal of “maximum recovery to the pre-injury status of burn survivors”. In addition to burns surgeons and nurses, physical and occupational therapists, rehabilitation nurses should also be included. The team may also include physiatrists, psychologists and psychotherapists, nutritionists, wound treatment professionals, social workers, and also patients [16] and their families.

Burn rehabilitation should be carried out by person with professional rehabilitation background. If possible, therapists can also be subdivided into physical therapists (PT), occupational therapists (OT), vocational therapists, orthotists and prosthetists. Otherwise, burn surgeons and nurses from burns units, who have received relevant rehabilitation training, can take over the duties of rehabilitation.

### Responsibilities

#### Burn surgeons

Burn surgeons are responsible for medical treatment of burn patients, including medications, life support, wound care and operations. They are the team leaders of the overall treatment plan during the acute and wound treatment period. Rehabilitation therapists should closely communicate with them about the time and treatments conducted during whole process [17].

#### Rehabilitation physician

To be a rehabilitation physician in a burn ward, experiences in wound care, surgical techniques and scar treatment are preferred. During the wound treatment period, burn rehabilitation physicians should develop a rehabilitation plan and confirm it with burn surgeons [18]. When wound closure is completed, burn rehabilitation physicians are responsible to work out the overall rehabilitation plan with therapists and supervise the plan’s implementation, to monitor the physical conditions of the patients and deal with comorbidities and residual wounds.

#### Rehabilitation therapists

Rehabilitation therapists provide comprehensive rehabilitation assessments, set short-term and long-term goals of rehabilitation, and implement the entire rehabilitation program according to the patient’s condition. Timely communications on the progress of the patient’s functional outcome to burn surgeons and rehabilitation physicians are required. For burn units without full time therapists, professionals from rehabilitation department of the hospital can be assigned for the job.

##### Responsibilities of PT

PT focus mainly on positioning, range of motion (ROM), muscle strength, endurance, balance, coordination and respiratory rehabilitation of the patients. They help patients regain the abilities of transfer, ambulation and proper gait. Various physical therapies can be used to eliminate or reduce the degree of dysfunction and improve mobility. The final goal of physical therapy is to enhance the adaptability of social participation, and improve the quality of life of the burn patients [19].

##### Responsibilities of OT

Responsibilities of OT are to maintain and improve ROM, muscle strength, endurance, flexibility, and coordination of limbs through designed target-oriented activities that burn patients can actively participate in, with the help of splints and scar treatment modalities. Restoring ADLs of burn patients and promoting social participation and reunification is the goal of the therapy [20].

#### Rehabilitation nurses

Rehabilitation nurses mainly coordinate between rehabilitation physicians and rehabilitation therapists [21], educate and promote knowledge of rehabilitation, provide guidance for positioning and ADL training, and facilitate patients to attain rehabilitation goal within limited time. They also provide guidance and supervision for the usage of pressure garments and splints. Moreover, they should recognize patients’ psychological changes and discuss them with physicians, rehabilitation therapists, and psychotherapists for further treatment [22]. Rehabilitation nurses play an indispensable connecting role among patients, their families, and the rehabilitation team [23].

#### Psychiatrists or psychologists

Psychiatrists or psychologists are responsible for assessing the patients’ psychological states and determining the need for medication, counseling, and other interventions to help burn patients overcome anxiety, depression, pessimism, and other psychological disorders after the injury, thereby helping burn patients establish good psychological adaptation to the injury.

## Assessments

Collection, quantification, analysis and comparison data of patients’ functional status with relevant information are foundations of functional diagnoses. Rehabilitation assessment is usually performed by using physical examination, instrument testing, clinical observations, and questionnaires to analyze and determine the functional status and potential of the patient.

To date, there are no globally accepted and standard assessment tools specifically designed for burn patients [24]. The widely used applications are as follows: 1) A goniometry for the measurement of ROM; 2) Manual muscle strength testing and grip dynamometer for the measurement of muscle strength; 3) Barthel index (BI) and the Functional Independence Measure scale (FIM) for the assessment of ADLs; 4) Vancouver Scar Scale [25] for the evaluation of scars; 5) Electromyography and nerve conduction tests for electrophysiological assessment of the neuromuscular system; 6) Exercise testing and pulmonary function test for the assessment of cardiopulmonary function; and 7) Psychological and mental disorder assessment scales.

## Rehabilitation therapies at different stages

The pathophysiological changes post-burn is clinically divided into shock, infection, and wound healing phases. Except for the accurate definition of shock phase, which is defined as 48 h to 72 h post-injury, these three phases overlap in time and interact with each other, therefore, there is no hard line between one another.

A concept that needs to be popularized is that burn rehabilitation starts day one after injury. It should be implemented before wound closure finishes and should not be a supplementary therapy afterwards, or patients may miss the optimal time frame for treatment and hamper the effectiveness of rehabilitation, which in turn will result in low compliances of treatments. Burn rehabilitation should begin immediately after injury and continue throughout the entire process until several months to years post-burn [26–28].

Rehabilitation is an indispensable part of the whole burn care process and need a multidisciplinary team approach [29]. It could be divided into the following two stages: wound healing and post-healing stage. In the wound-healing stage, burn surgeons are responsible for making various treatment decisions. Once wound closure is completed, rehabilitation should be coordinated and arranged by burn rehabilitation physicians and/or rehabilitation therapists.

According to the patient’s general condition, wound healing stage can be further divided into stages with unstable and stable vital signs. The two conditions are interconvertible. Post-healing stage can be subdivided into inpatient and outpatient rehabilitation stages [30].

### Therapies of patients with unstable vital signs

Patients are under a life-threatening situation during this phase. Therefore, therapies should be implemented carefully within acceptable range [31]. It mainly includes: 1) Appropriate positioning to reduce edema of limb and face; 2) Maintaining ROM [32]; 3) Splinting to keep joints in anti-contracture and/or functional positions; and 4) Communicating with and educating patients and their families to strengthen their confidence to therapies.

Prolonged immobilization may result in contracture of joints, which could be prevented or delayed by the following treatments: 1) Passive and/or active ROM training of intact and involved joints at least twice a day. During the treatment, duration, range and strength of individual treatment should be adjusted to a safe limit according to changes of vital signs (heart rate, blood pressure, and respiration rate) [33]; 2) To minimize the pain, therapies could be performed during wound debridement and dressing changes, if possible; 3) Contractures of tendon, collateral ligament and capsule can be minimized by appropriate anti-contracture positioning [34] and splinting (Table 1).

**Table 1:** Common contractures and anti-contracture strategy after burns

Body Part Burned	Common Contractures	Positioning and Splinting strategy
Neck	Flexion	Exercise every day, slightly extension position or splinting
Shoulder	Adduction	Exercise every day, abduction splints under arms
Elbow	Flexion or Extension	Exercise every day, alternate positioning strategy of extension and flexion
Wrist	Flexion or Dorsal Extension	Exercise every day, extension splinting of 20°
MP(Metacarpal Phalangeal Joint)	Hyperextension	Exercise every day, thumb opposition, 50-70° MCP flexion and IP joints in full extension using functional or anti-contracture splint
IP(Interphalangeal Joint)	Flexion	
Hip	Flexion	Exercise every day, fully extended and abducted, prone position if possible
Knee	Flexion	Exercise every day, extension splint
Ankle	Planter Flexion	Exercise every day, neutral position with dorsiflexion of 90°
Metatarsal-Phalangeal Joint	Dorsiflexion	Exercise every day, anti-contracture splint
Mouth	Microstomia	Exercise every day, mouth splints
Nostril	Stenosis of Anterior Naris	Appropriate dilator inserted into nostril

### Therapies of patients with stable vital signs

Vital signs are relatively stable in this phase, therefore, duration, range and strength of therapies could be increased according to patient’s tolerance. They are encouraged to participate in active movements. Therapies in this phase are: 1) Passive ROM training; (2) Active ROM and muscle strength training; 3) Edema control; 4) ADLs training based on patient’s capability; 5) Scar management; and 6) Preparation for returning to work [35, 36], school, and entertainment.

### Inpatient rehabilitation phase (post-healing stage)

During this phase, wound healing is completed and patient’s physical conditions are significantly improved. Patients are able to withstand relatively higher intensity of therapies. Thus, ADLs training should be focused on improving the overall capability. Therapies should be co-ordinated with the requirement of returning to their work, school, and entertainment.

The scar problems become prominent and comprehensive scar management would be extremely important. Rehabilitation in this phase includes the following: 1) ROM training, strength training, and gait training; 2) ADLs training; 3) Comprehensive scar management; and 4) Using toys and games to assist their rehabilitation processes for pediatric patients.

### Outpatient rehabilitation (post-healing stage)

In general, the most difficult time for burn patients is 1–2 years post-injury. Although patients have been discharged from the hospital, they still need long-term rehabilitation therapies and follow-up. Therapies in this phase include: 1) Making a follow-up plan; 2) ROM and strength training to improve physical function; 3) ADLs training; 4) Scar management; 5) Periodical assessments of functional status and adjust treatment plans accordingly; and 6) Considering reconstructive surgery if needed.

## Implementation of rehabilitation therapies

Burn rehabilitation physicians and therapists are responsible for assessing the patient’s functional status and making appropriate therapeutic plans for each patients.

### Positioning

Patients tend to maintain comfortable positions to avoid further pain. But positions of comfort are always the positions of contracture. Appropriate positioning is the first line and by far one of the best way to avoid contractures and dysfunction [37, 38]. Positioning should begin immediately post injury and maintain during the entire process. Positioning should be carried out together with proper ROM training, otherwise, a prolonged fixed position will also result in reduced ROM and contracture.

**Fig. 1:**
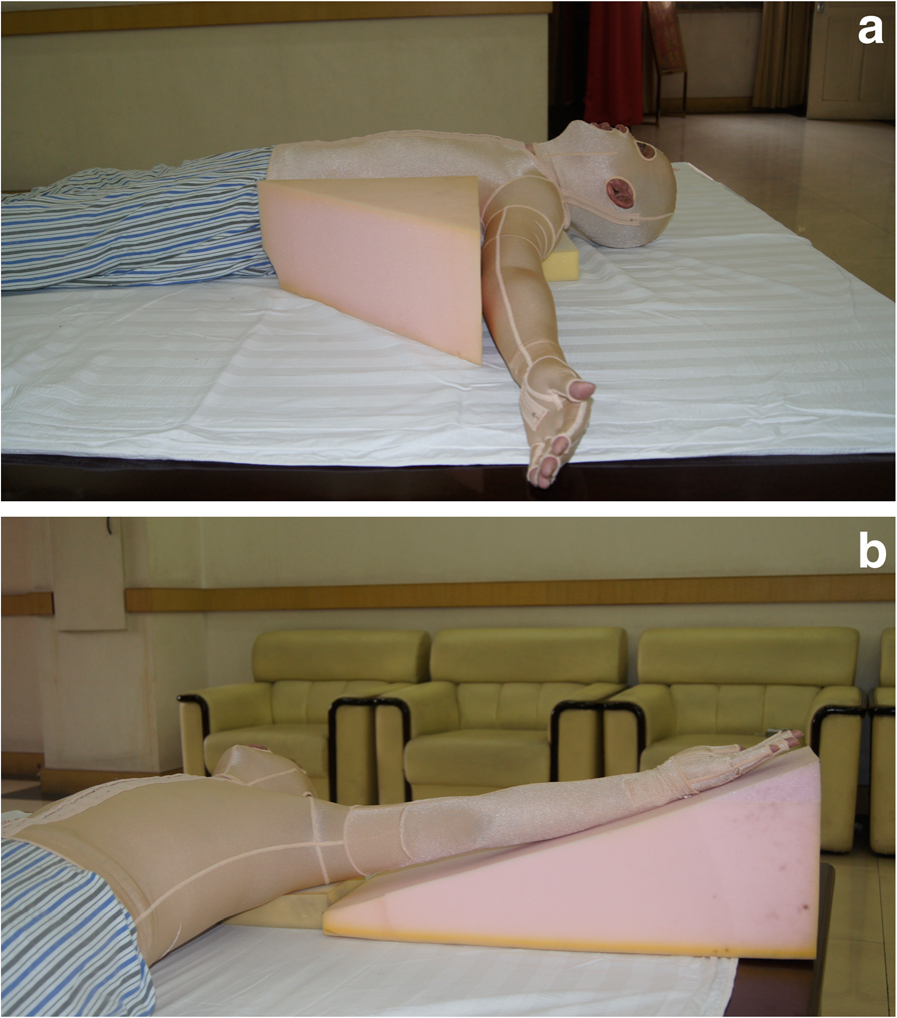
(**a**-**b**). Fully abduction with horizontal adduction of 15°–20° of the arms

**Fig. 2:**
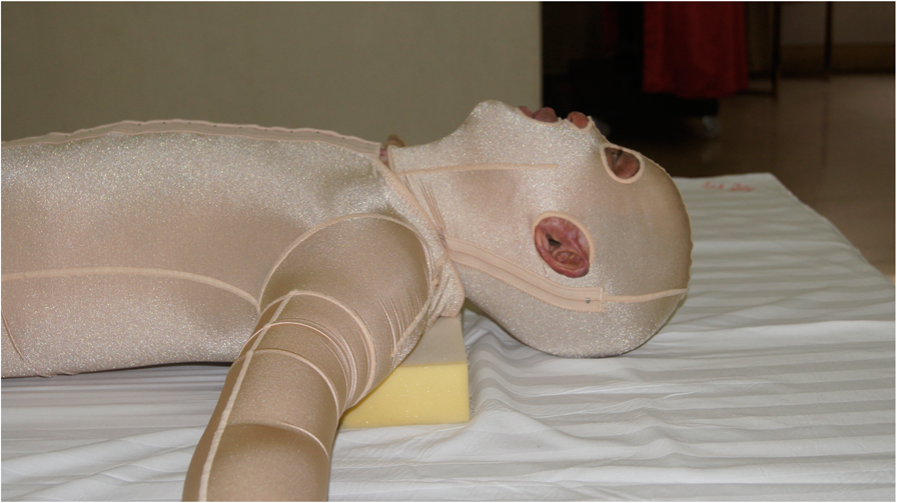
A pillow or cushion can be added under the shoulder to allow fully extension of neck

Positioning could be achieved through various modalities including pads, pillows, headboard, foam pads, splints and restraint belts. Here are some examples: 1) Mouth splint could be used for patients with deep burns around the lips during wound healing to prevent microstomia contracture; (2) Fully abduction with horizontal adduction of 15°–20° of the arms (Fig. 1) can prevent axillary contracture when wounds involve the upper limb(s) and the chest. Brachial plexus injury should be avoided by slight adduction of the arm; 3) Patients with anterior neck burns should avoid using pillows and maintain extension of the neck. A pillow or cushion can be added under the shoulder to allow full extension of neck (Fig. 2)

**Fig. 3:**
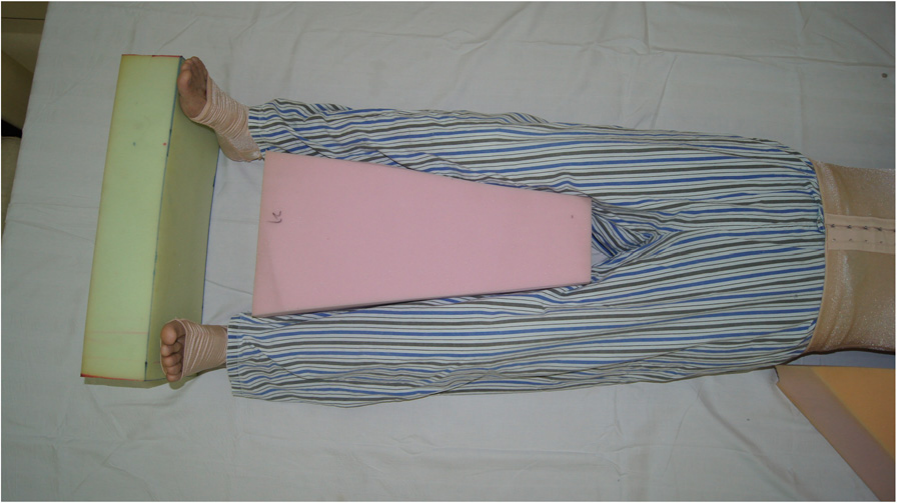
The hips should be kept fully extended and abducted (Figure 3) when wounds involve the hips and perineum

**Fig. 4:**
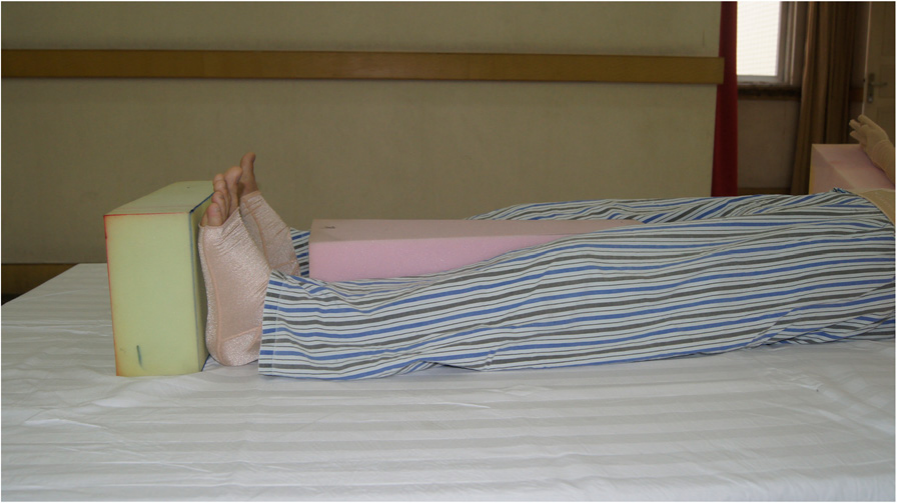
Foam pads or splints should be used to prevent planter flexion caused by Achilles tendon or scar contracture

. Patients with posterior neck burns should adjust the pillow to ensure that the neck slightly bends forward to prevent flexion contracture. Patients with bilateral neck burns should keep in a neutral position; 4) Patients with burns on the flexion side of elbow should place their elbow extended, while patients with burns on the extension side should maintain their elbow flexion at 70-90°. Circumferential elbow burns could adopt an alternate positioning strategy of extension and flexion. The forearm should be maintained in a neutral or supination position; 5) For wrist and hand, dorsal burns should be kept in a flexion position, while palmar burns should be kept in an extension position. Circumferential hand burns should maintain a functional or anti-contracture position. The position composed of thumb opposition, wrist slight extension, 50-70° MCP flexion and IP joints in full extension. All fingers should be separated with gauze to prevent web contraction. Splints can be used to maintain appropriate positions of limbs if necessary; 6) The hips should be kept fully extended and abducted (Fig. 3) when wounds involve the hips and perineum; 7) 10-20° flexion can be adopted using pads when burns affect the anterior knee. When burns are on the posterior side, the knee(s) should be maintained in extension. Splints can be used to keep the position if necessary; and 8) When wounds involve the ankle, it should be maintained in a neutral position with dorsiflexion of 90°. Foam pads or splints should be used to prevent planter flexion caused by Achilles tendon or scar contracture (Fig. 4)

.

### Therapeutic exercises

Therapeutic exercises are the basic and most important therapeutic strategy in rehabilitation medicine and include passive and active exercises. No special, complicated, or expensive equipment are needed but the exercise prescription depends on the expertise of therapists, who are skillful and capable of making correct diagnosis of patient’s functional problem. Therapists are responsible for developing proper plans to minimize injury and ensure effects during exercises.

Therapeutic exercises include: 1) Exercises to maintain ROM; 2) Exercises to enhance muscle strength; 3) Exercises to enhance endurance; 4) Exercises to improve coordination; 5) Exercises to restore balance; 6) Ambulation training; and 7) Exercises to improve cardiopulmonary function. Passive, active-assistant and active exercises, resistive exercises as well as stretching techniques could be used either alone or in combination based on patient’s condition.

Advantages and disadvantages of therapeutics should be weighted to avoid significant interference with patients’ general condition and clinical pathophysiological processes. Exercise prescription should be adjusted if 1) Unstable vital signs and existence of a life-threatening condition; 2) Presence of significant redness, swelling, heat, pain, and other signs of acute infection in the treatment area; 3) Therapeutic exercise may cause further tissue damage if necrosis, exposure of blood vessels, deep vein thrombosis, and bone fractures exist; 4) Immobilization is needed due to skin grafting, fracture fixation and other reasons; 5) If the patient has significant psychiatric conditions or unconsciousness, exercises might be impossible.

Exercises could start from major joints (with or without burn injury) using passive, active-assistant and active ROM training. The intensity needs to be adjusted based on the patient’s tolerance. Strict bed rest should be minimized and sitting out of bed and early ambulation with or without assistance should be encouraged as much as possible. All team members should be aware of that elevation and pressure bandaging can help to relieve the pain and edema during ambulation [39].

It is recommended to start exercises 5–7 days after skin grafting (or following the surgeon’s advice), active and passive ROM training at this time should be careful and gentle to protect the newly taken grafts. If the joint was not involved, ROM training can be conducted as soon as possible after the operation. Exercises and ambulation can also be carried out early if it will not affect the grafted area.

Active and passive ROM training after applying allograft and xenograft could start the first day post operation. Bandaging or splints could be chosen to immobilize the grafts for proper time according to the surgeons’ advice.

Exercises after artificial dermis transplantation could begin from unoperated limb(s) the first day post operation. Operated area should be bandaged or immobilized using splints. If joint was not involved, movement of operated limb(s) could begin 5–7 days post operation. If involved, time of exercises should be discussed with surgeons and rehabilitation physicians.

Exercises after sheet autografting could start after dressing change 5-7 days post operation. ROM training could be carried out according to the patient’s tolerance.

Exercises of donor sites could be introduced early post operation (post-operative day 1, if practical) using active and/or passive ROM training. Even if donor sites located on the lower limbs, patients could try to sit and walk with assistance, but should be careful with the grafted areas.

Intraoperative exercises (under anesthesia) can be introduced when discussed with or follow the decision from burn surgeon. ROM training and splinting can be easier under such circumstances, especially for children. Intraoperative ROM assessment could also be performed. But more carefulness should be paid to avoid tissue damage due to lack of protective reaction from patients under anesthesia.

Exercises under consciousness-sedation can be chosen for patients who cannot tolerate training even after giving medication or other pain control methods. Consciousness-sedation can be applied 2–5 days per week according to the judgment of anesthetist.

Hydrotherapy is performed to relieve itchiness, pain, and to improve patients' ROM and cardiopulmonary function. Different facilities could be used according to patient’s condition and specific situation of each burn unit. Some cautions include the following: 1) The entire process should be supervised by specialists such as therapists, nurses, or physicians in burn units; 2) Patients with open wounds should be treated very carefully to avoid cross infection as well as worsen of wounds or patients’ general conditions; and 3) Patients with unstable vital signs, or in infective status should not undergo hydrotherapy. The specific schedule of hydrotherapy should be decided by burn surgeon.

### Splinting

Splints are designed and fabricated by therapists or orthotists. Splints are tailored to help to maintain the functional or anti-contracture position of the injured body parts [40]. Application of splints requires a team work from therapists, rehabilitation physicians, burn surgeons, nurses, patients, and caregivers. The timetable of wearing splints as well as checking list of skin conditions could be stuck to the patient’s bedside. Any abnormal skin conditions caused by splints should be reported immediately to rehabilitation and clinical team. The intervals for monitoring vary from once every hour to once every 4–6 hours, depending on types of splints and skin conditions.

#### The continuous regimen

Splint is recommended to be worn continuously except for dressing change, skin examination and exercise [41]. It can be applied in the following situations: 1) To maintain or improve the outcomes of skin grafting, but the skin examination might be not practical with dressings; 2) To maintain the proper position of areas with circumferential, cross-joint, and deep burns; and 3) To retain the gained improvement of ROM.

#### The alternative application regimen

It is described as 10 hours on and two hours off. It can be applied under the following conditions: 1) To position areas with superficial circumferential or cross-joint burns; 2) To immobilize allografts and keep proper position; and 3) To maintain splinting as long as possible. When the splint is taken off, active and/or passive ROM should be carried out. However, if alternate application of splints significantly affects or limits active movements of the joint, the advantage and disadvantage of the splinting strategy should be carefully weighted.

#### Application only at night or rest

This strategy is mainly for patients who can perform daily activities with full ROM but still require maintenance of a position at rest.

#### Cautions

1) Closely monitor skin bruising, wounds appearances, and adjust application strategies accordingly; and 2) Timely adjustment of splints according to changes of patients' ROM.

### Comprehensive scar management

The chances of scar formation will increase if the healing process is over two weeks post burn. Scarring usually begins to develop within the first few months after burn, accelerates afterwards, peaks around 6 months, and will be stable and subside or “mature” around 12–18 months post injury. Active scars appear as red, raised and rigid with feeling like tight, itching and pain, as well as significant neovascularization [42, 43]. Hypertrophic scars around joints may hamper mobilization and result in deformity when contracted. To date, there is no single therapeutic strategy that can avoid hypertrophic scar formation completely. Combination of therapeutic strategies and interventions can achieve better outcomes [44]. Pressure therapy, positioning, splinting, ROM training, and therapeutic exercises are irreplaceable treatments, which can prevent, inhibit and improve scar proliferation and contractures, as well as soften scar and alleviate accompanied symptoms [45].

#### Pressure therapy

Pressure therapy is still the first-line treatment for scars, especially for those with deep burns [46]. It can relieve edema, inhibit growth of hypertrophic scars, promote scar maturation, protect the newly healed skin, and relieve itching and pain [47]. The most commonly used products include pressure garments, pressure pads, elastic bandages, rigid transparent facemasks, and splints [48].

The following are some notes for pressure therapy: 1) Pressure therapy is recommended for areas that healed 2–3 weeks post burn to prevent and inhibit scar formation. Areas healed over 3 weeks post burn, grafted, and donor sites of split-thickness skin grafts should receive pressure therapy. 2) Pressure therapy does not necessarily have to be postponed until wound healing is completed, and for areas that require more than two weeks to heal, pressure therapy could be attempted using elastic bandages overlay wound dressings, and always begin with lower pressure and check the wound healing process. 3) Pressure therapy and wound healing processes should be weighted to patients’ best benefits. For example, when pressure therapy hampers wound healing or causes skin lesions, lower pressure or shorter wearing time, and/or more frequent dressing changes should be considered. 4) Pressure therapy should be carried out progressively to reduce the chances of skin breakdown caused by friction or high pressure on newly healed, fragile skin, and to improve patients’ tolerance and compliance. If the newly healed skin is too fragile to tolerate higher pressure, elastic bandaging, with which the pressure can be easily adjusted, can be introduced as an alternative choice. 5) Pressure garments are recommended to be worn continuously over 23 h a day, only to be taken off when dressing changing, taking shower, and scar treating. Pressure therapy should be maintained until scar maturation, when the scar fades in color and becomes soft, flat and pliable. This process often takes 1–2 years or longer. 6) Therapists should monitor the conditions of pressure products regularly. As the elasticity would reduce, it should be replaced every 2–3 months. 7) For irregular or concave body parts, pads could be inserted to ensure the curative effect. 8) Pressure products can be used together with anti-scarring cream and silicone sheets [49, 50]. 9) Children should be closely monitored during the treatment because poorly fitted pressure products might cause severe malformation to the body parts.

#### Scar massage

Although no study has reported the exact mechanism of scar massage [51], application of deep and slow pressure to scars can help soften the scar and improve ROM, as well as relieve pain and itching [52, 53].

Scar massage has been widely recommended for scar treatment and may help in the following ways: 1) Scar is often dry and itching with ulceration and other problems, massage with cream and oil can help to moisturize and soften the scar, increase the pliability, help to relieve itching and pain. 2) The tightness of scar might be partly caused by excessive fluid retained inside. Deep and firm massage can help to resolve this problem. Exercises accompanied with scar massage can also help to increase ROM [52]. 3) Deep and circular massage can also help re-alignment of collagen fibers during scar formation. 4) Scar massage is also a way to desensitize newly healed skin and might promote sensory recovery.

#### Silicone sheets

Silicone sheets can effectively help scar softening and hydration [54]. Some patients might have rashes or feel itching during application; gradually lengthen the time may be better way to start with [55]. Evidence has shown that the application of silicone sheets alone has a certain anti-scarring effect [56] and better results can be achieved when combined with pressure garments [57].

#### Intralesional injection

Intralesional injection can be used to relieve symptoms and accelerate maturation and flatten of small hypertrophic scars, especially those with distinct itching and pain [58]. Currently, the most commonly used medications for injection are corticosteroids, and triamcinolone acetonide and betamethasone have been widely used. Although intralesional injection shows significant inhibition effects on scar formation and could accelerate scar softening and maturation, the treatment regimens are not unified, and there are various regimens derived specifically from each unit’s practice. Cautions are: 1) Patients should be fully and clearly informed about the possible therapeutic outcomes and side effects before the treatment; 2) It is strongly recommended that detailed records should be taken during the treatment, such as case history, scar imaging (digital photography), Vancouver Scar Scale scores, visual analogue scale (VAS) of pain and itching, episodes of side effects, and so on; 3) Localized, cosmetic-related scars as well as ones with significant itching and pain could be prioritized for injection; 4) The dosage given per injection should be limited and intervals of injection should be adjusted according to scar’s reaction and side effects of patients.

### Psychotherapy

Patients' attitude and motivation are important factors affecting the outcomes [59]. Psychological factors, rather than trauma itself, may have more profound impact on burn patients [60]. Each member of the burn team should pay attention to the psychological state of patients through daily communication [61].

Different psychological problems will be encountered in different stages of treatment: 1) During the acute and critical stage, vital signs are unstable and patients may exhibit anxiety, fear, hallucinations, and sleep disorders [62]. 2) As wound healing progresses, the demand of surgery and critical care reduces, while the intensity of physical and occupational treatments increases. Patients gradually realize the extent of the damage and potential impact on their future. They may develop depression and post-traumatic stress disorder (PTSD). PTSD affects approximately 30 % of burn patients [63], which might present with sensitivity, phobia, and sleep disorders. Medications and psychological consultations may improve the condition. 3) After the initial recovery and 1–2 years after discharge from the hospital, patients with physical limitations often suffer from emotional problems when adapting to family life and a new working environment [64, 65]. They may also be affected by PTSD and show various degrees of depression, which will be further aggravated if adequate psychotherapy is lacking or delayed. The psychological treatment of patients relies on long-term attention as well as the relationship between patients and psychiatrists. It is highly recommended for patients to receive psychotherapy from professional organizations if possible [66].

### Other types of physical therapies

Physical characteristics of light, electricity, ultrasound, magnetic field, water, paraffin, temperature, and pressure could be used to reduce local inflammation, relieve pain, improve muscle response, inhibit scar proliferation and accelerate blood circulation. Burn patients could possibly benefit from all those factors in inflammation reduction, wound healing [67], edema control, scar maturation and improvement of muscle and soft tissue conditions. The most commonly used are paraffin therapy, hydrotherapy [68], low-frequency electrotherapy [69, 70], medium-frequency electrotherapy, microwave therapy, shortwave therapy, air compression therapy [71], laser [72, 73], ultraviolet therapy [74], ultrasound [75], and cold therapy [76], which can be used alone or in combination according to the specific needs and conditions of the patients.

## The reintegration of burn patients

For burn patients, the road to return to their normal family and social life is very long and difficult [77], especially for those with disfigurements and dysfunctions. The whole team, including medical professionals, patients and their families, organizations [78] and government agencies [79], should be engaged in helping burn patients better adapt to their families and the society. Sports, entertainment activities, vocational training programs, burn survival groups and peer support groups [80], patient or family supporting groups [81], camps for burned children [82–85] and other similar programs might be helpful to burn patients as well as their families.
